# Differences in femoral component subsidence rate after THA using an uncemented collarless femoral stem: full weight-bearing with an enhanced recovery rehabilitation versus partial weight-bearing

**DOI:** 10.1007/s00402-021-03913-0

**Published:** 2021-05-21

**Authors:** Franziska Leiss, Julia Sabrina Götz, Matthias Meyer, Günther Maderbacher, Jan Reinhard, Lukas Parik, Joachim Grifka, Felix Greimel

**Affiliations:** Department of Orthopedic Surgery, Medical Center, Asklepios Klinikum Bad Abbach, Regensburg University, Kaiser-Karl V.-Allee 3, 93077 Bad Abbach, Germany

**Keywords:** Stem subsidence, Total hip arthroplasty, Cementless THA, Enhanced recovery, Weight-bearing

## Abstract

**Background:**

Femoral component subsidence is a known risk factor for early failure of total hip arthroplasty (THA) using cementless stems. The aim of the study was to compare an enhanced recovery concept with early full weight-bearing rehabilitation and partial weight-bearing on stem subsidence. In addition, the influence of patient-related and anatomical risk factors on subsidence shall be assessed.

**Methods:**

One hundred and fourteen patients underwent primary cementless THA and were retrospectively analyzed. Sixty-three patients had an enhanced recovery rehabilitation with early full weight-bearing and 51 patients had rehabilitation with partial weight-bearing (20 kg) for 6 weeks. Postoperative subsidence was analyzed on standing pelvic anterior–posterior radiographs after 4 weeks and 1 year. Subsidence was measured in mm. Anatomical and prosthetic risk factors (stem size, canal flare index, canal fill ratio as well as BMI and demographic data) were correlated.

**Results:**

Femoral stem subsidence rate was significantly higher for the group with an enhanced recovery concept compared to the group with partial weight-bearing at the first radiological follow up after 4 weeks [2.54 mm (SD ± 1.86) vs. 1.55 mm (SD ± 1.80)] and the second radiological follow up after 1 year [3.43 mm (SD ± 2.24) vs. 1.94 (SD ± 2.16)] (*p* < 0.001, respectively). Stem angulation > 3° had a significant influence on subsidence. Canal flare index and canal fill ratio showed no significant correlation with subsidence as well as BMI and age.

**Conclusion:**

In the present study, cementless stem subsidence was significantly higher in the group with enhanced recovery rehabilitation compared to partial weight-bearing. Small absolute values and differences were demonstrated and therefore possibly below clinical relevance. Anatomical radiological parameters and anthropometric data did not appear to be risk factors for stem subsidence.

## Introduction

Since its clinical introduction, hip joint replacement surgery is considered one of the most successful operations due to its high success and low complication rate [1]. One possible risk factor for early failure of total hip arthroplasty (THA) is the subsidence of the femoral stem [2]. Cementless THA in particular could be susceptible to subsidence [3]. Subsidence is defined as a distalization of the femoral stem in reference to the greater trochanter. According to the literature, the maximum of subsidence occurs within the first 6–8 weeks postoperatively [4–6] as bony ingrowth takes up to 4–12 weeks but it can also last up to 3 years [7, 8]. The risk of subsidence of the femoral stem before sufficient osteointegration is reported to be between 5 and 61.5% [9]. Reasons for subsidence are partly still unclear, but femoral stem design and type might play a relevant role as well as anatomical properties [3, 4, 6].

The canal flare index (CFI) and the canal fill ratio (CFR) are common criteria for describing proximal femoral anatomy and stem anchorage [4, 10]. Biomechanical studies have shown that a close proximal fit of the femoral stem optimizes the initial torsional stability [11–13]. An increased primary stability can lead to an improved bony ingrowth and minimizes fibrous ingrowth [14–16].

In accordance with previous recommendations, postoperative rehabilitation following cementless THA was performed with partial weight-bearing for 6 up to 12 weeks after surgery [17–21]. It was thought that early full weight-bearing might increase micromotion of the stem. The micromotion could result in fibrous ingrowth at the implant-bone-interface [22, 23]. Furthermore, partial weight bearing (PWB) might reduce the stress on the implant-bone interface and thus increases the probability of proper osteointegration and stable implant fixation. However, clinical studies supporting this theory were not conclusive [16, 18, 24–26]. On the other hand, several reports have indicated that early full weight-bearing after cementless THA showed no negative influence on implant stability [6, 25]. Tian et al. [27] were evaluating partial vs. full weight bearing (FWB) after THA with an increased femoral subsidence in the FWB group after 3 months but with no difference at a 2 year or later follow up. In recent years, an enhanced recovery concept after THA with full weight-bearing on the day of surgery has become increasingly established with no adverse effects [28, 29].

The present investigation was performed to evaluate the effects of different rehabilitation regimens on implant fixation. In this retrospective study, we compared the effect of early full weight-bearing with an enhanced-recovery scheme (ERP) on stem subsidence in comparison to partial-weight bearing (20 kg) after THA with an uncemented, collarless stem (Depuy Synthes collarless Corail femoral stem) in a radiological measurement. In addition, we asked, whether there are any anatomical and anthropometrical risk factors for stem subsidence. We hypothesized that early full weight-bearing with an enhanced recovery concept after THA shows higher stem subsidence in the follow up than partial weight-bearing after THA.

## Materials and methods

In the present retrospective study, 114 patients who underwent primary cementless, collarless THA between mid-2018–mid-2019 in a single centre were included. Inclusion criteria were primary THA using a DePuy Corail^®^ femoral stem due to primary or secondary osteoarthritis and the existence of a radiological data set with radiographs at the first postoperative week, at a first follow up after 4 weeks postoperative and a follow-up after about 1 year. The period was chosen because within this timeline the enhanced recovery setup was established and therefore both concepts could be ideally compared. Exclusion criteria were a malignancy of the femur or the pelvis, severe dysplasia of the hip, rheumatoid arthritis and a prior fracture.

The study was approved by the local Ethics Committee (approval number 20-2009-104). The study was applied in accordance with the ethical standards of the Declaration of Helsinki 1975.

Sixty-three of the 114 THA-patients received an enhanced recovery program with preoperative gait training and detailed lecture as well as non-steroid-anti-inflammatory-drug application just before the procedure. The operation was performed under spinal anaesthesia. Intraoperatively, tranexamic acid was administered topically and intravenously, local-infiltration analgesia was applied, no drains were used. Full weight-bearing was allowed right away. The patients of enhanced recovery (ERP) were mobilized for the first time 2–3 h after the operation with full weight-bearing. Furthermore, the patients received physiotherapeutic treatment twice a day during their hospital stay. Patients were instructed to use a newly established exercise circuit, which included a walking course, various muscle exercises and tutorials to improve coordination. The exercise circuit focuses on strengthening hip and knee muscles. Physiotherapy was administered by two specially educated fast-track physiotherapists. A treatment protocol for fast track THA was established. Physiotherapeutic treatment was performed under consideration of hip precautions.

The control group consists of 51 patients and received a conventional recovery program. The operation was performed under spinal anaesthesia in all cases. Preoperatively no NSAID was used. During the operation, no tranexamic acid or local anaesthesia was administered. Patients were instructed to walk with crutches with a load of 20 kg at each step of the operated leg for 6 weeks, accordingly to the traditional postoperative recommendations of the unit. During the hospital stay, patients received physiotherapeutic treatment once a day in consideration of hip precautions. After 6 weeks, patients were allowed to unrestricted weight-bearing. The patients were instructed for partial weight-bearing by physiotherapists and self-control by use of a body scale. In both groups, patients were discharged to a rehabilitation clinic on the seventh day after surgery in general. In our department, a standardized pain management concept was established regarding the recommendations within the WHO analgesic ladder [30]. The pain management was used for both groups equally.

The surgery was performed using an anterolateral approach (Microhip). In all cases, an uncemented, collarless DePuy Corail^®^ femoral stem with standard offset or high offset was used, according to preoperative radiological planning. The surgeons aimed for the maximal possible femoral stem size with the best possible bony support and rotational stability in all cases. The femoral stem was implanted using the same technique for both groups. The femoral medullary canal is opened with a box chisel. Then palpation of the medullary canal and preparation of the calcar. Broaching with the rasps is done manually until rotational stability is achieved. With the last raps, a trial position is made. An intraoperative X-ray was used in both groups after implantation of the trial femoral stem to confirm the correct fit and size of the prosthesis. In addition, the mobility is checked to confirm stability (90° flexion & internal rotation, adduction & external rotation). After dislocation and removal of the trial implant, the original implant is inserted. After repositioning the prosthesis, X-ray is made again. The DePuy Corail^®^ stem is a straight implant with a quadrangular cross-section that is made of forged titanium alloy. The neck-shaft-angle is 135° or 125°, depending on the offset variant. To prevent medullary obstruction, the corail stem has a tapered construct at the lower end. To improve primary mechanical stability the stem has vertical and horizontal grooves and the entire surface is coated with hydroxyapatite (HA). The thickness of the HA coating is 150 μm [31]. Furthermore, the HA surface ensures optimal osteointegration with the endostal surface to prevent fibrous fixation [31]. In all cases, a DePuy Pinnacle^®^ acetabular component (Depuy Synthes) was used.

Anterior–posterior radiographs of the pelvis were performed in a standardized standing position with centralized beam focus on the symphysis. Direct postoperative x-rays were performed on the 3rd–4th day after surgery in a standing position. All radiographs were examined digitally (MediCAD, mediCAD Hectec GmbH) by using the implanted femoral head size for calibration. The measurements were performed by two independent investigators (research assistant and surgeon).

**Fig. 1:**
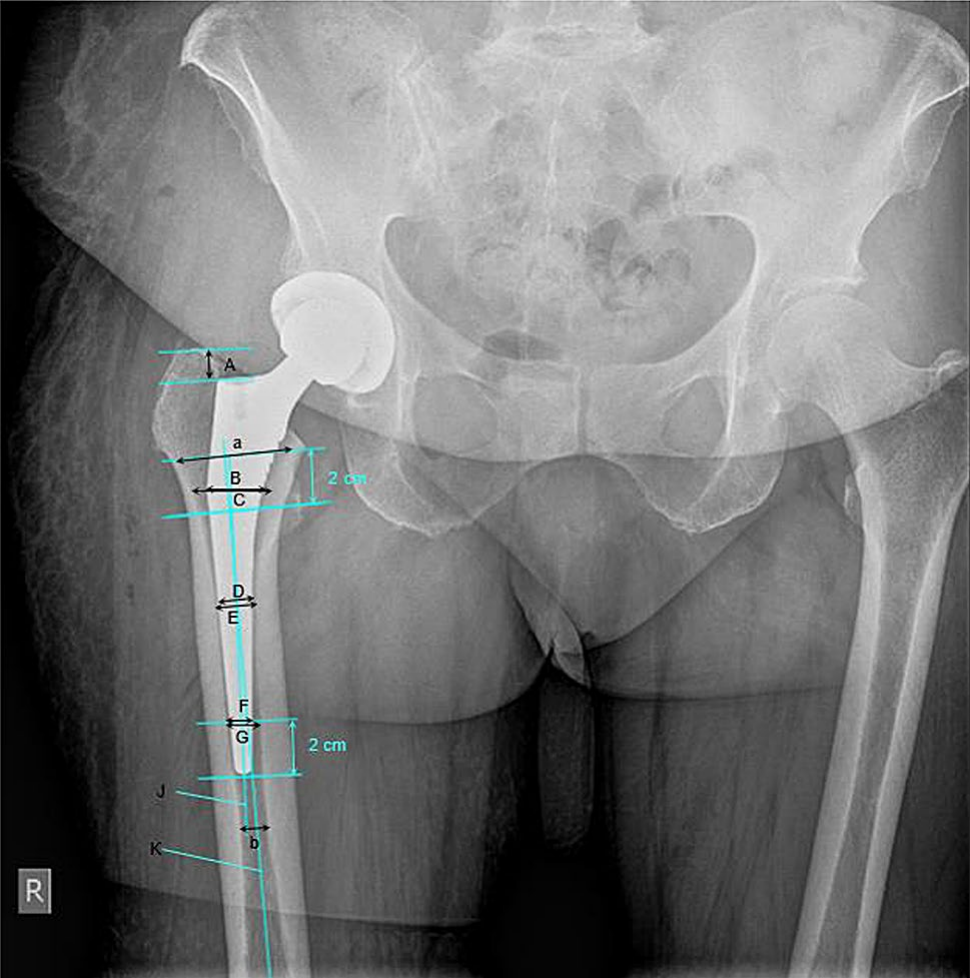
Measuring technique on anterior-posterior radiographs of the pelvis in standing position: subsidence, stem angulation, canal fill ratio and canal flare index

The following parameters were measured (Fig. 1):

1. Subsidence of the femoral stem was measured by comparing the immediate postoperative radiographs with the radiographs of the first follow up and/or the second follow up. The distance in mm from the greater trochanter to the shoulder of the femoral stem was measured by using parallel lines.
2. Varus or valgus stem angulation in reference to the long axis of the femur was measured on X-rays postoperatively.
3. Canal fill ratio at three different measuring points: at the distal third (2 cm above the stem tip), the middle third (between the measuring points of the distal and proximal third) and the proximal third (changeover of the proximal and distal stem part) of the stem on the radiographs postoperatively according to [4].
4. Canal flare index (CFI = a/b) was calculated by measuring the metaphyseal diameter 2 cm proximal of the middle of the lesser trochanter (a) and the isthmus diameter (b) according to Noble et al. [10].

Furthermore, other factors that could contribute to subsidence were evaluated: height, bodyweight, body mass index, age, gender and stem size. In accordance to Al-Najjim et al. [4] the extent of subsidence was grouped as followed:

group I < 3 mm, group II 3-5 mm, group III > 5 mm, group IV > 10 mm.

### Statistical analysis

For descriptive analysis mean values and standard deviation are given as well as the median and interquartile range (IQR). For comparison between the two groups, the Mann–Whitney *U* test was performed. A multiple linear regression was used to estimate risk factors for subsidence, the type of physiotherapeutic treatment as factor and BMI, Age, height, weight, gender, stem size, CFI or CFR as the covariates. A *p* value < 0.05 was considered statistically significant. No formal a priori sample size calculation was performed due to the retrospective (all available patients were included) and exploratory nature (no primary endpoint) of the study.

All analyses were performed using SPSS 25.0 (IBM SPSS Statistics, Armonk, NY – IBM Corp.).

## Results

General and demographic data are shown in Table 1. Median implanted femoral stem size was 13 (IQR 12;14) for the group of enhanced recovery (ERP) and 12 (IQR 11;13). for the group of partial weight-bearing. Mean subsidence for the enhanced recovery group showed 2.54 mm (0–9 mm, SD ± 1.86) at the first radiological follow up whereas the subsidence for the partial weight-bearing group was 1.55 mm (0–12 mm, SD ± 1.80). Mean subsidence was significantly lower for the group of partial weight-bearing (*p* < 0.001), Table 2. 28 patients of ERP had a subsidence < 3 mm, 21 patients a subsidence of 3–5 mm and 3 patients 5–10 mm at the first follow-up. No patient showed a subsidence > 10 mm. 43 patients of the group of PWB showed a subsidence < 3 mm, 3 patients of 3–5 mm and one patient > 10 mm. No patient had a subsidence between 5 and 10 mm at the first follow up, Table 3.

**Table 1:** General and demographic data, mean (*SD* standard deviation), median (*IQR* interquartile range) and percentage

	Enhanced recovery (ERP)	Partial weight-bearing (PWB)	*p* value
No. of patients	63	51	
Female: male	23:40	33:18	0.003
Age in years	61.5 (± 8.49)	68.7 (± 10.85)	< 0.001
BMI	28.1 (± 3.99)	29.3 (± 5.59)	0.187
Weight (kg)	87.6 (± 14.49)	81.6 (± 16.63)	0.043
Height (cm)	176.4 (± 8.53)	166.7 (± 8.05)	< 0.001
Stem size	13 (12;14)	12 (11;13)	0.45
Stem angulation > 3°	5 (7.9%)	5 (9.8%)

**Table 2:** Outcome data and analysis of proximal femur anatomy

	Enhanced recovery		Partial weight-bearing		*p* value
	Mean (SD)	Median (IQR)	Mean (SD)	Median (IQR)	
CFI	3.56 (± 0.57)	3.54 (3.15, 3.90)	3.71 (± 0.53)	3.67 (3.33, 4.08)	0.109
Stovepipe (*n*)	9		2		
Normal (*n*)	51		46		
Champagne-fluted (*n*)	3		2		
CFR (%) (proximal third)	67 (± 8)	67 (62, 74)	71 (± 21)	71 (64, 78)	0.904
CFR (%) (middle third)	87 (± 6)	87 (83, 90)	85 (± 8)	87 (79, 93)	0.608
CFR (%) (distal third)	91 (± 4)	77 (73, 87)	78 (± 11)	80 (69, 86)	0.636
Subsidence (mm) (1st follow up)	2.54 (± 1.86)	2.00 (1.00, 4.00)	1.55 (± 1.80)	1.00 (1.00, 2.00)	< 0.001
Subsidence (mm) (2nd follow up)	3.43 (± 2.24)	3.00 (2.00, 5.00)	1.94 (± 2.16)	2.00 (1.00, 2.00)	< 0.001

Mean (SD), median (IQR), level of significance < 0.05

*CFI* canal flare index, *CFR* canal fill ratio

**Table 3:** Subsidence of enhanced recovery group and partial weight-bearing group at the first radiological follow-up

1st follow up	Enhanced recovery (*n* = 52)	Partial weight-bearing (*n* = 47)
Subsidence (< 3 mm)	28 (44.4%)	43 (91.5%)
Subsidence (3-5 mm)	21 (33.3%)	3 (6.4%)
Subsidence (> 5–10 mm)	3 (4.8%)	0
Subsidence (> 10 mm)	0	1 (2.1%)

Mean subsidence at the second follow up was significantly lower (*p* < 0.001) for the group of PWB with 1.94 mm (0–15 mm SD ± 2.16) than for the group of ERP with 3.43 mm (0–10 mm, SD ± 2.24), Table 2. At the second follow up 23 patients of the group of ERP had subsidence < 3 mm, 30 patients had a subsidence between 3 and 5 mm and 10 patients 5–10 mm. Again, no patient showed a subsidence > 10 mm. For the group of PWB 39 patients had a subsidence of < 3 mm, 11 patients 3–5 mm and 1 patient > 10 mm. No patient had a subsidence between 5 and 10 mm, Table 4.

**Table 4:** Subsidence of enhanced recovery group and partial weight-bearing group at the second radiological follow-up

2nd follow up	Enhanced recovery (*n* = 63)	Partial weight-bearing (*n* = 51)
Subsidence (< 3 mm)	23 (36.5%)	39 (76.5%)
Subsidence (3-5 mm)	30 (47.6%)	11 (21.5%)
Subsidence (> 5–10 mm)	10 (15.9%)	0
Subsidence (> 10 mm)	0	1 (2.0%)

To date, no patient had revision surgery due to symptomatic subsidence. One patient in the ERP group had an early periprosthetic joint infection that required a surgical debridement, head and liner change. The patient received antibiotics for 12 weeks postoperatively.

The stem angulation > 3° showed a significant influence on stem subsidence in linear regression with *p* = 0.025.

Considering femoral anatomy as a risk factor for subsidence, canal flare index had no significant influence on subsidence (*p* = 0.109), Table 2. Most of the patients of the ERP group and the PWB group showed a “normal” canal flare index [32]. The canal fill ratio (CFR) showed no significant influence on subsidence at all three measuring points, (proximal third *p* = 0.904, middle third *p* = 0.608 and distal third *p* = 0.636), Table 2.

All other potential factors like BMI, height, weight, age and stem size showed no significant influence on stem subsidence.

## Discussion

Our data have shown that subsidence was significantly higher for the group of enhanced recovery than for the group of partial weight-bearing at the first radiological follow up after 4 weeks and the second follow up after 1 year (*p* < 0.001, respectively). Stem angulation > 3° showed a significant influence on stem subsidence (*p* = 0.025). Canal flare index (CFI) and Canal fill Ratio (CFR) showed no significant correlation to subsidence. Other factors like BMI, height, weight, age or stem size had no significant influence on subsidence.

Stem subsidence is considered as one possible factor for the early failure of THA. The risk of femoral stem subsidence prior to osteointegration is reported with rates of 5–61.5% in elective hip replacement surgery [9]. In recent years, enhanced recovery concepts after THA have been increasingly used to improve rehabilitation. To our knowledge, there have been no investigations on stem subsidence comparing an enhanced recovery concept and a conventional partial weight-bearing rehabilitation after THA.

The early and slight subsidence of a collarless, cementless femoral stem is thought to be an effect of impaction rather than true subsidence to implant loosening [5], as it can be related to an inadequate cancellous bone impaction intraoperatively. As the patient begins to weight-bear, the hoop stresses transmitted from the implant to the bone, compact the implant further, which leads to subsidence until the mechanical stability is achieved [4]. Subsidence within the first weeks of weight-bearing allows loading through the entire surface area of the stem which supports osteointegration and force transmission [4, 31]. Ström et al. [6] support this hypothesis. They observed early postoperative subsidence followed by stabilization of the implant. An error for radiographic measurement up to 2 mm is considered to be within the limits [33, 34]. Concerning clinical relevance, a subsidence up to 3 mm seems to be acceptable [35, 36]. Another factor for successful osteointegration is the degree of micromotion at the bone-implant interface. Micromotion of 150 µm or more is considered to lead to less stable fibrous tissue formation at the bone-implant-interface [16, 34, 37].

In the present study, most of the total subsidence occurred within the first radiological follow up after 4 weeks. At the second follow-up, both groups still showed a slight increase in subsidence. We explain the further increase of subsidence at the second follow-up due to the timing of the first radiological control after about 4 weeks. In literature it is described, that the maximum of subsidence occurs within the first 6–8 weeks postoperatively [4–6]. Other factors such as BMI, height, weight, age or stem size showed no significant influence on subsidence. Similar results are seen in the study of Schiffner et al. [38] However, a closer look at data reveals that the ERP group had a lower BMI and younger age than PWB group.

Campell et al. [3] have shown in their radiostereometric analysis (RSA) study a mean subsidence of 0.58 mm (range − 0.23–3.71 mm) for the collarless cementless DePuy Corail^®^ stem after 2 years. The authors reported subsidence to be confined to the first 6 months following THA. In the follow up of 13 patients after 14 years no further subsidence was monitored [39]. Ström et al. [6] used an uncemented Zimmer CLS^®^ stem and compared early unrestricted weight-bearing to partial weight-bearing. RSA analysis showed 1.2 mm (+ 0.11–6.76 mm) subsidence at 24 months in both groups with no difference in the migration pattern. Most of the subsidence occurred within the first two postoperative months. Also, Selvaratnam et al. [5] and Al-Najjim et al. [4] found most of the subsidence occurring within the first 6 weeks after surgery.

Both RSA-studies of Ström et al. [6] and Campell et al. [3] have shown a lower subsidence rate than our collective. Our results are comparable to the study of Ries et al. [40]. They reported a subsidence rate for collarless cementless stems of 3.1 mm (± 2.8) at 6 weeks follow up.

Ström et al. [6] have shown no significant difference in subsidence between the unrestricted and partial weight-bearing group after a follow up of 24 months. The group of unrestricted weight-bearing was instructed to full weight-bearing, hip flexion, extension and abduction as soon as tolerated. In our collective, the patients of ERP were mobilized for the first time 2–3 h after the operation with full weight-bearing and received intensive physiotherapy 2 times a day.

Other potential factors for stem subsidence are anatomical conditions such as canal flare index (CFI) and canal fill ratio (CFR). CFI has wide variations and is divided into “stovepipe”, “normal” and “champagne fluted”. Ishii et al. [41] investigated the proximal femoral anatomy and CFR in an Asian population. It has been observed that canal flare index was significantly larger in hips with failed osteointegration than in those with successful osteointegration. Furthermore, suboptimal changes were seen in a larger distal fill with a smaller proximal fill and a narrow femoral canal, which could lead to an unfavourable long-term clinical outcome. In contrast, the study of Cooper et al. [42] observed that patients with a smaller or “stovepipe” morphology of the proximal femur tended to be at risk for failed osteointegration. The authors mentioned that as stem size increases, the smooth distal portion of the stem increases in relative width compared with the proximal coated portion. These larger stems therefore also tend to have a greater degree of canal fill than the mid and distal thirds, leading to distal rather than proximal wedging and fixation [42]. The subgroup analysis of Ries et al. [40] showed a significant difference in subsidence for “champagne-fluted” femora compared to "normal" fluted femora with 3.6 mm and 2.8 mm (*p* = 0.015). But canal flare index did not significantly influence subsidence. In the present study, most of the patients had a “normal” CFI (ERP + PWB group, *n* = 97). There is no significant influence of CFI to subsidence in our investigation. All patients of ERP and PWB group with subsidence of group III or IV (5 mm or more) had “normal” fluted femora.

In our data, CFR of the proximal third, middle third and distal third showed no significant difference between ERP and PWB. Furthermore, we found no correlation of CFR (neither proximal third, nor middle third or distal third) and subsidence. In our collective CFR at the proximal third was 67% for ERP and 71% for PWB and at the distal third 91% for ERP and 78% for PWB. Ishii et al. [41] found the proximal CFR to be 69.1% for successful and 62.8% (*p* = 0.02) for failed proximal osteointegration. At the distal third Ishii et al. [41] observed a CFR be 90–100%. Cooper et al. [42] observed a canal fill at the mid-third for failed osteointegration of 95% and at the distal third of 97% in comparison to successful osteointegration at the middle third with 85% and 81% at the distal third. The data of Ries et al. [40] showed a CFR at the distal third of 80% for collarless stems. Thus, there is some variation in the different studies regarding the CFR with partly divergent results.

This study has several limitations like its retrospective study design. The retrospective analysis with a radiological follow up limited the number of cases, as cases with residence far away or patients from abroad did not consult our outpatient clinic for a regular follow-up. Furthermore, there was no follow-up for clinical outcome parameters and a correlation with stem subsidence. The clinical hip function was not assessable. Possibly biased selection of cases by the surgeons and the individual surgical approach of implant fixation could as well have influenced our measurements. Regarding the demographic and general data, there were some significant differences between the groups, which is due to the retrospective nature of the study. However, we consider the significant differences in height and weight to be negligible, as there is no significant difference in BMI. In addition, our investigation results are restricted to a single implant design. Furthermore, the maximum follow up period in our study collective was about 12 months, so long-term results regarding early implant failure are not available. So far, no revision surgery was necessary due to an early implant failure. The significant results regarding subsidence between PWB and ERP might not show clinical relevance, which should be evaluated in the context of longer follow-up periods. Due to the advantages of the enhanced recovery concept as a shortened convalescence and a faster functional recovery without increased mortality or morbidity as well as a reduction of the length of stay, we apply it as the main treatment in our department and use partial weight-bearing only in exceptional cases. Future prospective studies, possibly comparing different femoral components, must demonstrate the long-term effects of early femoral stem subsidence in an ERP and PWB setup.

## Conclusion

In the present study, subsidence was significantly higher in the enhanced recovery group compared to the partial weight-bearing group, using a collarless cementless femoral stem (DePuy Corail^®^), possibly below clinical relevance regarding the minor absolute values and differences. Most of the subsidence was seen in both groups at the first radiological follow up after about 4 weeks. In the following period, until the second follow up, a subsequent minimal subsidence occurred. Stem angulation > 3° showed a significant influence on stem subsidence. Anatomical parameters such as CFI and CFR did not represent risk factors for subsidence. Other factors such as BMI, weight, age and stem size showed no significant influence on stem subsidence as well. Further randomized controlled trials with large cohorts are required to identify the problems of subsidence in detail.
